# Wait for Me: Chronic Mental Illness and Experiences of Time During the Pandemic

**DOI:** 10.1007/s10912-023-09829-7

**Published:** 2023-12-26

**Authors:** Lindsey Beth Zelvin

**Affiliations:** School of English, Division of Arts and Humanities, University of Kent in Canterbury, Canterbury, UK

**Keywords:** Mental illness, Chronic illness, Narrative, Temporality, Medical humanities, Pandemic

## Abstract

As someone diagnosed with severe chronic mental illness early in my adolescence, I have spent over half of my life feeling out of step with the rest of the world due to hospitalizations, treatment programs, and the disruptions caused by anxiety, anorexia, depression, and obsessive–compulsive disorder. The effect of my mental health conditions compounded by these treatment environments means I often feel that I experience time passing differently, which results in sensations of removal and isolation from those around me. The global shutdown caused by the COVID-19 pandemic seemed a way for normative bodies to experience the passing of time the way I always have. In this paper, I extend Dr. Sara Wasson’s analysis of the ways in which chronic pain resists narrative coherence to my own temporal experience of chronic mental illness, specifically my embodied experience of the pandemic. I use that embodied experience as a case study for examining how the reciprocal nature of time and narrativity, as outlined by Dr. Paul Ricoeur, can create isolation for those struggling with their temporality due to chronic mental illness. To acknowledge and grapple with the ramifications of discursive and material privilege involved in such situations, I include an analysis of Robert Desjarlais’s 1994 article “Struggling Along: The Possibilities for Experience among the Homeless Mentally Ill,” in which he investigates a similar phenomenon of being outside of *structured sequential narrative time *in the residents of a Boston shelter for the mentally ill.

## Introduction

The COVID-19 pandemic has changed the way many of us relate to the world. It brought about a level of global anxiety I had never witnessed in my lifetime. And in the midst of that initial anxiety, when the world around me shut down and my family and friends sheltered in place, I found solace. This engendered complicated emotions, causing me to grapple with questions the answers to which I had no clear way of uncovering. How could I find relief in something that was causing such pain for the rest of the world? And why didn’t the anxiety and fear affect me the same way they seemed to affect others? In the early days of the pandemic, I attempted not to dwell on this. I journaled, I read, I spent time with my family, and I tried to ignore the nagging guilt I felt for how this global disaster had brought me a semblance of peace I had not felt in a long time.

As someone who was diagnosed with anorexia, anxiety, depression, and obsessive–compulsive disorder early in adolescence, I have spent over half my life feeling out of step with the rest of the world. For two years, I was treated as an in-patient, a partial hospitalization patient, and an out-patient, attending treatment programs and missing months of school while trying and failing to adjust from the regimented schedule of psychiatric wards to the freedom of the outside world and back again. The effect of my mental health conditions, compounded by these treatment environments, means I often experience time passing differently, which results in sensations of removal and isolation from those around me. What I mean by this is that I struggle to remain within what I will term *structured sequential narrative time*. In practice, this entails dissociation and separation from both my body and my present circumstances—as if I’m watching myself on a screen or through a thick glass wall, there but not there as I am consumed by a paralyzing combination of anxiety and detachment, inundated by intrusive thoughts and unable to produce the necessary coherence to participate in the world around me. When the lockdown came, it was like a weight had been lifted off my shoulders; I didn’t have to participate in the world around me and, in fact, could not do so even if I wanted to because that world seemed to have stopped turning.

As I embarked on my doctoral research a year and a half later, my mind kept traveling back to the beginning of lockdown and searching for ways to unpack the emotions following me into this next chapter of my life. Not dwelling became unsustainable. Instead, I began to interrogate. In their book *Autoethnography: Understanding Qualitative Research*, Tony E. Adams, Stacy Holman Jones, and Carolyn Ellis write, “Often, research projects begin with events that turn *us*—our thinking, feelings, sense of self and the world—and *others*—our friends and families, members of our social, political, and cultural communities, and others who are different from us—*inside-out*” (Adams, Holman Jones, and Ellis 2015, 47; original italics). The pandemic seemed to turn the world inside out, and I felt I could not move forward until I found a way to make sense of what it did to mine.

The pandemic, to me, felt like this great equalizing force was operating. It was the first time I can remember ever feeling completely on pace with the people around me because we were all living without a clear sense of futurity. For once, I did not have to worry about fighting with time because we were all standing still. While the relief was immense, it was quickly followed by the immense guilt of contemplating its cost. This became further complicated as communities began to emerge out of lockdown and move forward into a post-pandemic world. I was seized by grief and anxiety upon watching the world around me start to move again. I felt frozen and isolated, afraid of the time I had lost, the present I could not fully take part in, and the future I now had to shape. But I didn’t know how to explain this to anyone, and I feared trying, which further separated me from the people in my life. It is this process I interrogate within the contents of this paper, using my lived experience of the pandemic as a case study for examining the way in which chronic mental illness can lead to non-linear, non-narrative experiences of time. My research builds on the theory of the reciprocal nature of time and narrativity outlined by Dr. Paul Ricoeur in his essay “Narrative Time” while also examining how that different experience can create a sense of isolation for those who find themselves outside of *structured sequential narrative time*.

I draw on Dr. Sara Wasson’s research on ethical witnessing of pain and suffering to illustrate the way in which physical and mental illness can disrupt a person’s—in this case, my own—sense of temporality. In her article, “Before Narrative: Episodic Reading and Representations of Chronic Pain,” Wasson (2018, 107) writes that “illness is often described in terms of a narrative crisis, being locked in a present without a sense of a coherent narrative of past and imagined future.” Being “locked in the present,” she argues, causes immense difficulties when trying to narrate experiences of chronic pain, as sufferers are trapped in the immediacy of crisis and unable to produce a coherent narrative arc for their experience. Wasson’s focus in this paper is identifying the ways in which chronic pain resists narrative coherence while working towards a methodology for ethically and authentically rendering these inchoate experiences. I found her work exceptionally resonant with my own experience as a person with chronic mental illness. The phrase “locked in a present without a coherent narrative of past and imagined future” put words to experiences I had never been able to articulate. Even before my hospitalizations and the disruption brought on by the pandemic, I often found myself unable to remain focused on the present while equally unable to escape it, willing moments to move faster and days to move slower as I feared wasting my life in this invisible box in which I had somehow placed myself. This sensation caused my first panic attack at nine years old; I became physically ill at the thought that I was not enjoying a father-daughter camping trip as much as I should have due to my inability to ground myself in the present the way I thought I was supposed to.

My doctoral research chronicles and investigates the process of writing my personal narrative of chronic mental illness through documentation of my lived experience as a patient, writer, and academic to build a methodology for telling these stories. The first year of my study focused on critical research in narratology and the medical humanities, specifically narratives of illness, to build a framework for my hybrid memoir that will straddle the borders between personal narrative and critical interrogation of the methodological, ethical, and literary responsibilities involved in crafting such a narrative. In doing this research, I realized that many of the difficulties I have encountered in trying to tell the story of my experience come from being unable to reconcile my experiences of time with the people and narratives around me. This creates a lack of narrative coherence in both my life and my writing. Strangely, it was this feeling of being *out of time* that made it easier for me to adjust to the unprecedented disruption caused by the pandemic. And in speaking to others in my life who struggle similarly with chronic illness while also immersing myself in the fields of medical humanities, disability studies, and narratology, I came to realize that my experience was not as singular as I had thought.

Many theorists within the medical humanities have examined the way in which illness affects a person’s capacity for temporality and narrativity. Angela Woods, in her essay “The Limits of Narrative: Provocations for the Medical Humanities,” discusses how the privileging of narrative can further marginalize those dealing with illness, encouraging the reader to question “whether narrative should remain the privileged form for the interpretation or restitution of that self-experience [of illness]” (Woods 2011, 11). In a later article, “The Recovery Narrative: Politics and Possibilities of Genre,” she and her co-authors Akiko Hart and Helen Spandler, offer a critique of the recovery narrative as a genre, raising concerns about the way in which it can “occlude those stories and silence those voices which do not fit its strict parameters” (Woods, Hart, and Spandler 2022, 14). S. Kay Toombs also investigates how the temporal dimension of illness can impact a patient’s ability to communicate their experience in her article “The Temporality of Illness: Four Levels of Experience.” And Arthur Frank’s *The Wounded Storyteller: Body, Illness, & Ethics* looks at illness as an inherently postmodern experience, claiming “the anything-but-tidy conventions of postmodern memoir—its lack of linearity and competing voices—fit experiences that are interrupted” (Frank 1995, 70–71). I refer to these theorists throughout this paper and mention them here to situate my inquiry within a critical context. However, Wasson and Ricoeur remain the primary focus of this article, as their resonance with my lived experience provides an opportunity for me to close read my own experience of temporality before, during, and after the pandemic from an autotheoretical perspective. As this paper relies on both my embodied experience of chronic mental illness and my academic journey of finding ways to articulate that experience authentically, the use of autotheory, a term which “refers to the integration of theory and philosophy with autobiography, the body, and other so-called objective modes” is essential to its objectives (Fournier 2021, 18).

Throughout my life, I have read my own experiences through the work of other writers and theorists. A part of living with chronic mental illness that is not discussed enough is the way in which one’s own perspective becomes devalued through usually well-meaning interventions by family, friends, and medical professionals. Having been diagnosed at fourteen, I have spent nearly half my life being told that I often cannot trust my own brain. The way I chose to deal with this uncertainty was to gain expertise over the experiences I was not trusted to articulate. I became a researcher, a writer, and an academic. I turned my disability into my vocation. And it has been the greatest privilege of my life. But many do not have such opportunities. My material and discursive privilege have allowed me to seek treatment for my chronic mental illness and pursue research that I find valuable to both my own personal growth and the growth of the field. That privilege also affected the way I was able to deal with the pandemic, during which I sheltered in place with my family without having to choose between safety and financial security. I feel it is important to acknowledge this privilege early and often, as this paper documents only one experience: my own. In a later section, I will discuss an article by anthropologist Robert Desjarlais titled “Struggling Along: The Possibilities for Experience among the Homeless Mentally Ill,” in which he investigates a similar phenomenon of temporality in the residents of a Boston shelter for the mentally ill. While Desjarlais’s account of the residents strongly resonates with my own experience of mental illness and incarceration, the circumstances of the people described in his article are very different from mine. I include this discussion of their lived experience to demonstrate the ways in which discursive and material privilege can drastically impact the circumstances of those with very similar temporal experiences. I hope this will demonstrate the possible applications and extensions of this research beyond studies of the pandemic into more specific studies regarding experiences of chronic mental illness across varying demographics.

## Representing the Ineffable: Stepping Outside of Traditional Narrative Structures

Of illness, Arthur Frank (1995, 56) writes, “In the beginning is an interruption. Disease interrupts a life, and illness then means living with perpetual interruption.” Traditional narrative structures can struggle to capture the experience of this perpetually interrupted life due to their dogged drive toward restitution and resolution. These continual interruptions stand in direct opposition to the achievement of the satisfactory conclusion they seek. To examine the difficulty inherent in representing these interrupted experiences, I return to Dr. Wasson’s article. She proposes “to avoid marginalising vulnerable voices…we need a complementary critical stance less attentive to the narrative arc of a text—and as such less attentive to an individual’s ‘personal illness journey’” (Wasson 2018, 106). The “personal illness journey” to which she refers can be seen all over what Wasson calls the *survivor genre* of illness memoir, but she also makes a point to highlight the way in which even “medical humanities scholarship [which] has long challenged such triumphalist narration … celebrate[s] particular narrative typologies and certain ideal temporal orientations” (Wasson 2018, 107). Even Frank himself spends a large part of *The Wounded Storyteller* describing the “three narratives that storytellers and listeners use to structure and interpret stories, respectively: restitution, chaos, and quest” (Frank 1995, xiv). While Frank discusses the strengths and pitfalls inherent in each narrative typology in detail, his work still assumes the primacy of narrative as the way in which to convey these experiences. In a similar vein, Wasson describes the expectations of a successful illness text at the beginning of her article. Chief among them is narrative coherence in both the self and the story that the self is attempting to tell. But that level of coherence requires the protagonist to be able to place themselves securely within time and to be able to convey that temporality within their writing. This is where the difficulty arises for both Wasson and me.

People dealing with chronic physical and mental illness can find it difficult to remain within *structured sequential narrative* time due to their own physical and mental health, the environments they live in, the cultures they are part of, and the institutions they rely on for support. I use the term *structured sequential narrative time* to denote an experience of temporality that is narratively coherent and linear, in which one event seems to logically follow its predecessor without disruption, pause, or dissociation. Under my definition, a person living within *structured sequential narrative time* is usually able to actively engage in the present moment, following it to its conclusion and into the next present moment, creating a linear and narratively coherent sequence of events. Conversely, a person living outside of it can find themselves stuck in the perpetual present Wasson describes, dwelling within certain moments unable to escape while finding other moments invaded by past traumas and future anxieties. For those struggling, time becomes a difficulty to grapple with rather than a natural process to experience.

This difficulty is exacerbated by the cultural expectations of our medical institutions, in which being a good patient means being easily diagnosed, treated, and cured. Frank refers to this as the “restitution narrative,” writing that “contemporary culture treats health as the normal condition that people ought to have restored” (Frank 1995, 77). He describes it as the narrative that “dominates the stories of most people I talk to, particularly those who are recently ill, and least often the chronically ill” (Frank 1995, 77). The perpetual interruption that is the life of a chronically ill person seems to defy the restitution narrative, subverting the expectations of the medical system. This inadvertently places the patient and the medical professionals who seek to help them on opposing sides. Dr. Wasson examines this phenomenon in the specific context of chronic pain, arguing that it “disrupts the assumptions of our ‘analgesic culture’ that expects pain to be diagnosable and remediable” and puts those dealing with it in “a liminal position, with the social peril that implies” (Wasson 2018, 107). These words chimed with my experience of chronic mental illness, as I repeatedly experienced the frustration of doctors, therapists, psychiatrists, social workers, and teachers when my “issues” were not neatly and easily resolved. Both the chronically physically ill and the chronically mentally ill deal with complex, recurrent conditions that require a level of continuous care in a society where often the only two understandable options are to recover or die. Western society in general—and Western medicine in particular—are deeply uncomfortable with those who occupy the in-between due to the remission-flare-remission nature of chronic illness. In occupying that liminal space, they become transgressive figures, isolated from the world around them. Wasson (2018, 107) writes that “many people living with chronic pain report that they are excluded, marginalized, and disregarded” due, in part, to the way in which they “flout the narrative conventions to which illness experience should conform” (i.e., getting better or dying).

This has, in many ways, been my experience. After two years in and out of hospitals for my anorexia, I came out of treatment at almost 16, expecting and expected to be *recovered*. But I was far from it. My body was no longer in danger, but my mind was in chaos, and this took a massive toll. It was nearly a year before I managed to carve out a new normal as the person I had become, developing a routine designed to keep me safe and (relatively) well, which included therapy, an inclusive learning plan, medication, and other necessary accommodations. It took a relapse and several more years before I accepted that my mental illness was a condition I would live with for the rest of my life and that while I could employ strategies to manage it, I would never be rid of it. This is when I entered “the remission society,” a place in which “the foreground and background of sickness and health constantly shade into each other” (Frank 1995, 9). I was a person who, while no longer life-threateningly ill, would never be entirely well. Many of my family and friends still have difficulty with this concept, good-naturedly telling me that I am not mentally ill but have simply had to deal with some hard times in my life. This attempt at reassurance comes from the fact that the idea of chronic mental illness is one with which a lot of people struggle to come to terms. From their perspective, I am a smart, friendly, high-achieving young woman who has had some emotional disturbances. Because I can move through society as a *normal* person—maintaining relationships, progressing in my career, holding entertaining conversations—I cannot be mentally ill, as I do not fit their image of what mental illness looks like. I might have fit that description in my disastrous teenage years, but I have moved *beyond* that now, and many of these people are more than willing to attribute my past issues to traditional teenage angst. They are unable to reconcile how the woman they see before them talking about her doctoral research could have so much trouble accomplishing simple tasks like brushing her teeth and getting out of bed in the morning.

What is difficult for them to understand is that my issues are not contained to those few years of crisis and hospitalization. I was dealing with them long before anyone realized what they were. I have been fighting with time (and losing) for as long as I can remember. I have always longed for what was past, wishing to go back, feeling deeply uncomfortable in the now and uncertain about the future. I remember sitting in bed with my mom after my fifth-grade graduation, sobbing, as I talked about how now I was going to middle school and soon it would be high school, and then I would have to leave for college, and I was not ready for college; I could barely handle sleepovers. My mom laughed, wrapping her arms around me as she promised that all those things were a long way away and that, when they did come, I would be ready. She was partly right; I was absolutely ready to leave home by the time college came around, but not in the way I had imagined.

Growing up happened both too fast and too slow for my liking. I learned too early about my parents’ (and my own) powerlessness in the face of my mental illness. Within a year, the illusions I had of my parents knowing what to do in the face of danger and crisis were shattered. I watched people I loved do their very best to save me from myself, and I watched them fail. I watched them get angry and lash out in desperation, reaching the end of their rope, time and time again, only to find more because they didn’t have a choice. I was their daughter, they loved me, and they had to keep trying. But I could not help but fear that one day they would have to stop, that they would have no more to give. Sometimes, I wonder if I hadn’t gotten *better* when I did, if maybe they would have. It is one of the most terrifying feelings in the world to fear you might fall so far that the people who love you most would have to give you up for their own sanity.

My illness came at one of the most influential transition stages of life: early adolescence. The time when one is supposed to start learning how to take responsibility and control over one’s own life. That development was derailed by my illness. By the time I was *better* enough to start life again, I felt like I was too far behind. Everyone seemed to have moved on without me. I had gone through so much in such a short amount of time that I felt irrevocably separated from the people who were supposed to be my friends and peers. I was “reentering a world that [could] not imagine, and [did] not want to imagine [the] dissolution” of self I had experienced (Frank 1995, 107). No one could understand, and I did not have the words to explain it to them. I have been trying to figure out how to do so ever since. Even now, nearly a decade later, I still feel two steps removed from those around me. And the loneliness, isolation, and fear that comes with that feeling can often be more painful than the actual conditions with which I have been diagnosed. It separates me from the rest of the world, putting me in a situation where I must either pretend to be okay at the risk of my own health or attempt to explain (and often justify) an experience I do not fully understand to people who have no frame of reference. For people with chronic illness, continuously living through an experience that does not conform to normal expectations of narrative coherence, we find ourselves pushed to the borders, dealing with the pain of our conditions as well as the resulting exclusion. Wasson calls this position “*the temporality of thwarted connection*,” explaining:

The term seeks to convey the experience of a present in which one reaches for connection—for diagnosis, medical care, emotional support or companionship amid acute suffering—while aware of the (justified) anticipation of imminent failure and future pain, the recollection of past failures and past pain, acute self-awareness of one’s present performativity … and one’s ongoing somatic and emotional distress. (Wasson 2018, 109)

I tried to write through this distress, hoping that if I could make sense of what was happening to me on the page, I could present it to others in a way they would understand, and I would no longer be so alone. But I kept coming up against the same obstacle. In a journal entry written just after my relapse seven years ago, I wrote:

You can’t write your life story, or any story for that matter, if there is no point. As human beings we have this incessant need for meaning, for closure. Nobody wants to read the story of a person just going about their daily life. Existing. Surviving. There has to be a purpose. In order for a chronicle of my life to be intriguing in any way, it must possess one of three endings: I come to a life altering realization that changes the course of my future for the better, I find acclaim and success in my field despite my shortcomings which makes my life outwardly meaningful, or I die. People read stories about people with mental illness for the ending. They either get better, they change the world, or they die. A story about me navigating the constant ups and down that come with depression, without ever hitting rock bottom or breaking the cycle has no draw for anyone. Because there is no closure. There is no ending to tie a neat little bow around and say it’s done. Humans need to believe that everything has a meaning. That eventually things will make sense. That we will understand and finally have closure. That is what we believe as children and it is the belief we cling to as adults.

Looking at this now, I find it interesting how apt my description of the problems posed by trying to write about chronic mental illness within a traditional narrative framework was, even if I did not quite understand the reasons behind it yet. At this point, I was nineteen and just beginning my second year of university. I had a bit of experience in literary theory and none in the medical humanities, so this writing comes solely from the way in which I experienced living through and attempting to record my struggles with mental illness. In the coming years, I would work towards a more specific, nuanced, and academic perspective of this issue, but it would not come fully into focus until I read “Narrative Time” by Paul Ricoeur. Its relation to the illness experience would be further illuminated upon reading “The Temporality of Illness: Four Levels of Experience” by S. Kay Toombs, in which Toombs uses phenomenology to “provide the key insight that illness is experienced … in a fundamental way as a temporal entity” (Toombs 1990, 228).

In “Narrative Time,” Ricoeur argues for the close relation and interconnectedness of narrative and temporality. He writes, “The story’s conclusion is the pole of attraction of the entire development … Looking back from the conclusion to the episodes leading up to it, we have to be able to say that this ending required these sorts of events and this chain of action” (Ricoeur 1981, 170). He makes the point that the reader’s investment in the story, driven by the way in which the story pulls our attention from one point to the next, leads them to develop expectations and, therefore, requires a story to be “followed to its conclusion … [which] rather than being predictable … must be acceptable” (Ricoeur 1981, 170). In other words, we require from our endings the pay-off of our beginnings and middles. The conclusion of a story must follow logically from its preceding events, fulfilling our requirement for narrative coherence. It must not only conclude the story but *complete* it, and thereby bestow meaning on the previous events through their contribution to the narratively coherent whole. When this requirement is unfulfilled, our disappointment may be unparalleled.

Chronic illness often prohibits not only a satisfactory conclusion but any conclusion at all. The illness is cyclical rather than linear, making any attempt to narrate it fraught with difficulty. Ricoeur (1981, 174) writes, “To tell and to follow a story is already to reflect upon events in order to encompass them in successive wholes.” In the case of chronic illness, we are unable to reflect upon these events because we are still living through them and will continue to live through them indefinitely. Frank refers to this difficulty in his description of the chaos narrative, writing that “those who are truly *living* the chaos cannot tell it in words” (Frank 1995, 98). What then happens to our stories? Our lack of traditional temporality does not make them any less valuable. In fact, in many ways, it increases their importance as the representation provided by such stories is of a kind infrequently articulated and desperately needed. Wasson (2018, 106) writes that “people enduring chronic pain are often oddly invisible, with healthcare practitioners, kin and employers failing to recognise the severity of their experience.” Her article is designed as a response to this failure, as “part of a wider project seeking to expand the critical vocabulary around the analysis of chronic pain representation” (Wasson 2018, 109). I take this project a step further to extend Wasson’s research beyond representations of chronic pain to illuminate the temporal experience of chronic mental illness, specifically my own embodied experience during and “after” the pandemic.

## Unpacking the Understanding and Relief Brought by the Pandemic

Ricoeur (1981, 177) writes that “the primary direction of care is toward the future. Through care, we are always already ‘ahead of’ ourselves.” This resonated strongly as a person with generalized anxiety disorder. To me, there is only a slight distinction between *care* and *stress*. My life is spent worrying about all the things that might happen as a result of all the things that have happened, which causes me to miss out on all the things that are currently happening, something I will worry about once the present has become the past. This cycle results in a panic response, in which I freeze or implode, either of which makes it difficult to go about my daily life as a functional member of society. I find myself unable to act due to the fear of what is, what has been, and what may be.

Ironically, this fear further removes me from *structured sequential narrative time*. In “Narrative Time,” while discussing the role of narrativity in the different levels of temporal organization, Ricoeur (1981, 167) writes that “a story is *made out of* events to the extent that plot *makes* events *into* a story” and “places us at the crossing point of temporality and narrativity.” The concept of a story being made up of events is not new; we all learn this at a very young age as we begin to discover what stories are: hearing them from our parents at bedtime, reading about them at school, and even beginning to tell them ourselves. Stories are often our earliest way of making sense of the world as we attempt to understand the way in which we relate to it. But events require presence and participation from those involved. If stories are made up of events that must be enacted, what happens when one is unable to do so?

In Ricoeur’s view, it is our ability to act that puts us at the first level of temporality, which he calls “within-time-ness,” claiming:

It is the phenomenon of intervention, in which our powers of action are linked to the world order, that what could be termed the structure of intersection characteristic of within-time-ness is constituted, in the nether zone between ordinary time and true historicality. (Ricoeur 1981, 173)

In other words, our agency is what places us within time and gives us the ability to interpret it, both for ourselves and others. This is further illustrated in Ricoeur’s (1981, 173) statement that “in the instant of acting, when the agent seizes hold of such circumstances and inserts his or her action into the course of things, the temporal guides provided by the chain of meaning attached to manipulable objects tend to make world time prevail over the time of action.” Action places us within the time of the world in that our ability to act is directly tied to our ability to experience time as part of the world around us. But the difficulty of chronic illness, both physical and mental, is that it makes it far more difficult to act.

When Ricoeur speaks of events, he writes that each individual event “receives its definition from its contribution to the development of a plot” (Ricoeur 1981, 167). Ricoeur’s use of plot implies that a story requires a level of narrativity to be valid. If plot “places us at the crossing point of temporality and narrativity” and an event “receives its definition from its contribution to the development of a plot” it is possible that the person who lacks narrativity due to the repeated interruption of chronic illness can become disconnected from their own temporality and therefore isolated within time. If people, as narrative creatures, take meaning from the stories we tell and create for ourselves, then it could be argued that being unable to turn the events of our life into a coherent narrative results not only in the absence of a fully realized story but also in the events themselves being stripped of meaning due to their lack of contribution to the development of a plot. In this vein, I have often found myself not only isolated and lacking a story to tell myself during flares of my chronic mental illness—which include depressive episodes, prolonged periods of intense anxiety, panic attacks, and increased eating disorder behaviors and relapses—but having immense difficulty maintaining any meaning I have managed to find in the individual events of my life, meaning which is often tied to and reinforced by the “recovery narrative” I was taught to strive for in the early stages of my illness. Angela Woods and her co-authors write in “The Recovery Narrative: Politics and Possibilities of Genre” that one of the genre’s “defining feature[s] … is the establishment of a particular relationship between narrator and reader … in which the narrator is positioned as seeking recognition from the Other that the knowledge they possess about their own experiences qualifies as ‘insightful’” (Woods, Hart, and Spandler 2022, 230). By the parameters of such a genre, I must be able to convey my experiences in such a way that “Others” who have never had such experiences will regard me as “insightful.” This means I must find a way to convey experiences resistant to traditional narratives through such structures or risk being viewed as an unreliable narrator of my own experience.

This is a heavy burden to carry and compounds the difficulties of chronic illness while also not providing the necessary support to the person suffering. What is often most desired and comforting in such situations is connection: the sense that one is not alone in one's pain, that others stand beside them even if those others cannot fully understand what the individual is living through. Stories of such experiences, or “chaos narratives” to use Frank’s term, “are hard to hear” as “the teller is not understood as telling a “proper” story” and “the anxiety these stories provoke inhibits hearing” (Frank 1995, 97–98). People tend to be afraid of what they do not understand, and their reaction to such discomfort may be to turn away or try to force these stories back into traditional narrative structures. But Frank (1995, 101) writes that “the challenge of encountering the chaos narrative is how not to steer the storyteller away from her feelings. … The challenge is to hear.” Wasson supports this claim, suggesting that “what these narrations [of chronic pain] often seek is not someone to understand the specific nature of the pain, but rather to acknowledge the reality of the suffering” (Wasson 2018, 111). Someone to witness and validate their embodied experience, to say, as Wasson (2018, 111) writes, “I believe you suffer and I stand beside you.”

In reading Wasson’s article, I realized that this connection was what I experienced at the beginning of the pandemic. In March of 2020, I was in a very bad mental space. I was experiencing frequent panic attacks at my place of work, struggling to do what was needed to progress in my field, and feeling exceptionally isolated. When I heard that everything was shutting down because of COVID, the relief was overwhelming, as I realized that I would not have to go back to work and fight down my anxiety attacks; that my lack of progress in my research did not matter considering everything was put on hold anyway; that my parents would be home with me; that my sister would come home from university; that I would not feel so alone anymore. It was like the pandemic came at the perfect time to give me everything I needed to make me a part of the world again—albeit a contained, safe, domestic world.

I tried to keep this feeling to myself, understanding that, for most, this experience was not a relief but an unprecedented crisis. We have all lived through traumatic world events—in my lifetime alone, there’s been 9/11, Hurricanes Katrina, Sandy, and Ida, the disastrous 2004 Indian Ocean earthquake and tsunami, and countless mass shootings (I grew up in America, meaning this became a sad but not unexpected fact of life)—but the kind of extended, liminal experience created by the pandemic was something new to most. Time seemed to stand still as we were asked to stay inside our houses and wait. For many, this level of helplessness and sense of being stuck in a perpetual present was a completely new sensation, causing a variety of responses. It created high levels of fear, anxiety, uncertainty, and restlessness.

The social aspect of the pandemic is especially illuminated by Dr. Wasson’s discussion of affect theory. She writes that “much affect theory examines the complex ways in which the present moment is shaped by suffering” (Wasson 2018, 108). Though degrees of suffering differed greatly depending on the situation and circumstance, the pandemic created an inescapable sense of collective trauma. Wasson (2018, 108) writes that the scholarship of affect theory “is concerned with the inseparable entanglement of the somatic, the social and … the emotional … [and] seeks language to describe emergent, visceral, often inchoate forces.” She quotes theorist Raymond Williams in discussing his notion of “structures of feeling,” claiming that they “are partially affective in that they involve ‘a social experience which is still in process, often indeed not yet recognised as social but taken to be private, idiosyncratic, and even isolating’” (Wasson 2018, 108). This description of an evolving affective experience resonates with my experience of the early days of the pandemic: sheltering in place with my immediate family, waiting in a perpetual present for the situation to unfold. We suffered individually, processing our own emotions as best we could, isolated in our own little bubble. But this very experience of isolation and fear as we sat within this global uncertainty is what connected us to the rest of the world in ways we did not yet have the words or temporal distance to understand.

As a person used to living in a state of continuous anxiety who has spent most of her life struggling with time, this level of uncertainty was not new for me. While the state of the world was objectively disconcerting and worrying, my personal experience of relief came from the fact that it finally seemed like everyone else was feeling what I had always experienced. This was further illuminated upon reading S. Kay Toombs’s “The Temporality of Illness,” in which she emphasizes the way in which “the temporal constitution [of illness] is important in understanding the manner in which illness is *lived through* by the patient” (Toombs 1990, 228). Toombs (1990, 237) writes that “since the immediate experiencing of time is in no way comparable to an objective accounting of time, physician and patient constitute the temporality of illness differently,” but in the case of those early days of the pandemic, everyone around me seemed to be stuck within their own “immediate experiencing of time.” Therefore, rather than being pushed to the margins by my experience of chronic mental illness, I found myself at an advantage because I could deal with the uncertainty of this “suspended and unpredictable site” better than most of the people around me (Wasson 2018, 108). I was used to what Wasson (2018, 108) refers to as the “labour of incoherence, of inhabiting a cryptic present moment” because I had been doing it for years. And I had the added solace that, in this case, I was not doing it alone.

Ricoeur (1981, 169) writes that “the existential now is determined by the present of preoccupation which is a ‘making-present,’ inseparable from awaiting and retaining.” He illustrates this by explaining the relationship between the term *now* and the objective measurement of a day, writing, “A day is not an abstract measure; it is a magnitude which corresponds to our concern and to the world into which we are thrown” and that in this case “saying ‘now’ becomes for us synonymous with reading the hour on the face of a clock” (Ricoeur 1981, 169–170). “As long as the hour and clock are still perceived as derivations of the day that link concern with the light of the world,” Ricoeur (1981, 170) writes, “saying ‘now’ retains its existential significance.” Toombs also addresses this issue, calling it “a radical distinction between lived time and objective time, or between inner and outer time” (Toombs 1990, 229). It is this distinction, she argues, that creates the disconnect between patient and physician when describing the temporality of the illness experience, as the conditions under which the patient *lives* this experience make it difficult for them to measure it in the way the physician requires. In both cases, the problem arises when saying “now” becomes “a form of the abstract representation of time” or when “the ‘now’ of pain appears to be endless” (Ricoeur 1981, 170; Toombs 1990, 232). We are placed within time by our relationship to the objective measurements of the world, such as the day and, by extension, the clock. If we take this a step further, we could argue that it is often our routines that create for us a sense of being within the time of the world. Going to work, going to school, running errands, seeing friends, these are things that place us within time. When those routines are interrupted, when we become stuck in a perpetual present, our sense of time becomes muddled. Ricoeur (1981, 169) writes, “It is because there is a *time to do* this, a right time and a wrong time, that we can reckon *with* time.” For many people, this is what the pandemic removed. But for those of us who never really had it, we found ourselves in a situation where the people around us could, at last, have a frame of reference for the world we had been living in for years, one in which “illness-as-lived is experienced as an ever-present, enduring consciousness of disorder which resists measurements in terms of objective time” (Toombs 1990: 237).

## “Experience” or “Struggling Along”? Vulnerable Populations in Less Privileged Situations

Sara Wasson (2018, 107) argues that narrative activity and the capability to produce a narratively coherent self are privileged activities, writing that “scholars of class, feminism and postcoloniality have identified many ways in which a narratively coherent self is a cultural construction imbricated with privilege.” This privilege of narrativity “requires not just a particular sense of a temporally enduring self, but an active drive or tendency towards form-finding, story-telling, and revision” (Woods 2011, 9). This is a lot to expect from those in the thick of the illness experience Toombs describes, where “time seems to ‘stand still’ in that past and future coalesce into a stagnating present” (Toombs 1990, 237). To further engage with the real-world implications of privilege in regard to experiences of temporality and narrativity, or lack thereof, I turn to Robert Desjarlais’s 1994 article “Struggling Along: The Possibilities for Experience among the Homeless Mentally Ill,” an anthropological study of the residents of a homeless shelter for the mentally ill in Boston.

The shelter where his ethnographic study takes place was set up to “provide temporary housing for persons troubled by mental illness,” Desjarlais (1994, 889) explains. To gain a bed in the shelter, a person must be double marginalized by both homelessness and a mental health diagnosis; Desjarlais names schizophrenia and bipolar disorder in what appears to be an attempt to convey the necessary level of severity. While his description of the behaviors and struggles of the patients at points reminded me of my own experience, the very fact that I can process it as an *experience*, as well as the circumstances under which I became ill and the way in which my illness was handled by those closest to me, puts us in very different positions. I find this—my discursive and material privilege—is what most differentiates me from the shelter’s inhabitants, rather than our diagnoses, and I find it interesting that these two most “severe” and recognizable diagnoses are the ones he references by name, as nowhere in the article does he record any of the inhabitants diagnoses or mention any of them discussing a diagnosis with him. Whether or not this was intentional on the part of Dejarlais, it speaks to stereotypes regarding certain types of mental illness and the ability to function “normally.” I would argue that given Desjarlais’s own descriptions of the shelter’s inhabitants, their lack of material and institutional support was a greater disadvantage than whatever their diagnoses may have been. This would explain why the illnesses of schizophrenia and bipolar disorder are only mentioned at the beginning of the article and never referenced in connection to any of the specific patients with whom Desjarlais interacts.

The “temporary housing” of the shelter is continuously reinforced by the staff, as they make it clear through words and actions that it is not a home. They maintain this through the use of discipline, requiring shelter inhabitants to follow rules in order to remain in the shelter and removing them, either for an hour or a night depending on the severity of the infraction, when these rules are not followed. This repeated emphasis on the impermanence of their situation, as well as their powerlessness within it, only serves to reinforce the timelessness of the inhabitants, as they are put in a situation in which they possess no agency, forced to survive in the best way they can by disconnecting from the world around them in order to preserve their place in what is often, while only a temporary solution, their best option.

The worst option in this scenario is “the street” where many of these people have come from. Desjarlais (1994, 890) writes that in his discussions with the shelter inhabitants who have come from “the street,” they refer to it “as if it were a single location with a singularly forced sensorium of cold weather, fear, anonymity, transience, and a constant, unsettling tendency to get on one’s nerves.” “The street” puts people in the increasingly untenable situation of being both vulnerable and invisible, causing many to lose a sense of their own personhood. In this situation, the safety of the shelter is preferable. But maintaining that safety requires them to distance themselves from triggers which may cause them to behave in ways that could get them kicked out. Desjarlais (1994, 891) writes that “for many, there is a common and pragmatic need to keep the sense within equatorial lines—to seek comfort and safety in the routines of shelter life, and so spend one’s days in a way that skirts the fears, worries, and afflictions that impinge.” This technique is effective for maintaining survival within the shelter but makes it nearly impossible for inhabitants to connect with the world and with each other. They live outside of time, within fleeting moments to which they are highly sensitive. In their struggle for balance, most inhabitants maintain a level of distance from those around them, taking solace in the fact that there are others around but avoiding any close connections that could result in a disruption of stasis. “The stasis,” Desjarlais (1994, 896) writes, “tends to grow more fundamental the longer people stay in the shelter,” meaning that the very technique that allows the residents to survive can also keep them trapped in this impermanence indefinitely.

While Desjarlais examines the cultural construction and potentialities of the term *experience* among the shelter’s inhabitants, rather than those of narrativity, much of what he discusses is relevant in both contexts, and he acknowledges this in his research. His understanding of experience has much in common with Ricoeur and Wasson’s views on narrativity, especially his interrogation of the assumption that experience is a basic fact of being human. “Experience,” he writes, “is a crucial element of contemporary academic thought; to try to write about humans without reference to experience is like trying to think the unthinkable. Yet despite its apparent necessity, as something that can and must be thought, its universality remains in question” (Desjarlais 1994, 886). His claim that “the problem with taking experience as a uniquely authentic domain of life is that one risks losing an opportunity to question the social production of that domain and the practices that define its use” evokes Wasson and Ricoeur’s arguments about the constructed nature of narrativity and the ways in which assumptions of its being a natural condition of humanity can isolate those who are incapable of producing coherent narratives (Desjarlais 1994, 887). In addition, his definition of experience reads similarly to Ricoeur’s description of “within-time-ness” as the first level of temporality. It seems to be this “within-time-ness” that Desjarlais finds missing among the residents of the homeless shelter.

Desjarlais (1994, 887) suggests that “experience is not an existential given but rather a historical possibility predicated on a certain way of being in the world” and that his goal is to “understand what conditions are necessary if people are to experience or, alternatively, to struggle along,” a phrase he mines from a response of one of the shelter’s residents to his question about how she is doing. Like narrativity and being within time, Desjarlais (1994, 887) argues that “today … experience is largely rooted in individual agency” as “a person ‘has,’ ‘learns from,’ or ‘discloses’ an experience.” Experience, it seems, requires a certain level of narrativity. Without it, there is existence but no connection, creating a lack of being-in-time, a lack of meaning, and a disconnect from the world in which that existence takes place.

Desjarlais (1994, 888) argues that experience, in this context, “possesses hermeneutical depth” and “readily equates with a person’s inner life or consciousness,” connecting it to the “Western genealogy of the self” in which “experience is often seen as the foundation of human agency.” Lack of experience, then, directly connects to a lack of agency, which results in a lack of narrativity as the “transient, episodic succession of events” remain disparate, unmemorable pieces of a perpetual present rather than building into a linear, coherent plot (Desjarlais 1994, 888). Desjarlais ends his introduction with the acknowledgment that there is an assumption within the modern West that “we can only grasp our lives through narrative” and that this same ideology often applies to experience, as “the idea that experience accumulates in time through stories builds chiefly upon the relation between forms of life and narrative orderings of time” (Desjarlais 1994, 889). If this is the definition of experience, he argues, then what happens to people for whom experience is not a possibility? This is what his study seeks to understand.

Desjarlais emphasizes that though “the disabling troubles of mental illness surely play a role” in whether a person “experiences” or “struggles along,” “it is a set of specific political, social, cultural and environmental forces that leads people” to one or the other (Desjarlais 1994, 897). This helplessness in the face of impermanence, as well as the lack of privacy and agency, contributes to “an episodic orientation toward time, with each incidence taking place over any larger temporal context” (Desjarlais 1994, 897). So while mental illness can be what puts people in the kind of situation that leads to them needing to make use of a shelter, the amount of time spent within it and the sense of invisibility, isolation, and powerlessness produced by its impermanence is often what causes the residents to remove themselves from being-in-time and therefore what causes them to “struggle along” rather than “experience.”

My incarceration was very different, as I was placed in verified treatment programs by my parents with the expectation of coming back to my daily life once I was *better*. But even my hospitalizations—which ranged in length from days and weeks in the psych ward to two-and-a-half month stays at two different residential treatment centers—affected the way in which I was able to be-in-time. I often found myself going into survival mode in these situations, coping by pacing, fidgeting, and repeating phrases and numbers over and over to myself in the same way Desjarlais describes the residents of the shelter behaving. I was also alternately comforted and irritated by my fellow inmates, craving a certain level of connection while also wanting to keep them at arms-length. I hated the sense of being imprisoned but appreciated the safety of a low-stakes daily routine. The difference between my incarceration and that of the residents Desjarlais writes about is in the level of care received and the expectation of recovery rather than in how we handled our incarcerations.

It makes me wonder how they dealt with the pandemic.

## Conclusion: Processing the Interruption and Attempting to Move Forward

Ricoeur (1981, 175) writes that “the plot’s configuration … superimposes ‘the sense of an ending’ … on the open-endedness of mere succession.” Narratively, we expect a satisfying conclusion to the successive events we experience; they cannot just come one after another but must build into a greater whole. There must be an overarching moral to the story for our discrete experiences to have resonance. And so I notice that as I attempt to move forward from the massive interruption of the pandemic, I find myself surrounded, and nearly suffocated, by Frank’s restitution narrative. As I watch those closest to me work to return to the status quo, I am overcome by an all-consuming drive to push forward and make up for the time lost, to make myself productive and successful in spite of this interruption, as if it never happened in the first place. It feels nearly impossible to give myself the space to stop and account for the ways in which that lost time impacted me.

When the vaccine first became widely available and restrictions began to lift, I felt more in crisis than I had over the course of the previous year in and out of lockdown. Coming to terms with the fact that, after all the chaos, I was back to where I had been at the start, except more time had passed and I was even further behind, was painful and frightening. Through no fault of my own, I had lost even more time. The discourse around pandemic productivity making its way around social media made me feel even worse, as if I should have been spending this interruption accomplishing something rather than simply existing, surviving, and “struggling along.” As an over-achieving twenty-something, the pressure to make something of myself had always been intense, but now it was crushing. I felt added pressure to catch up as everyone around me seemed to be moving forward at warp speed. There was no time to waste, or process, or grieve what had been lost.

I feel these ramifications as I move forward in my post-pandemic life. And I am one of the lucky ones in that I can mask my vulnerabilities (to an extent) to move within society, even if I am not fully in-time. Many do not possess that capability. This is why I have chosen to examine my own experience, as I am lucky enough to have the discursive privilege to do so. Arthur Frank (1990, 109) writes that “responsibility implied by an experience of chaos cannot be exercised from within the chaos.” While I do still struggle with my chronic mental illness, I have, for the moment, moved outside the chaos, and therefore it is my responsibility to articulate my experience in order to give voice to those who cannot. Because I know more than most that “being a mute witness, caught within the chaos itself is a condition of horror” (Frank 1990, 109). To be left alone in a perpetual present of crisis, especially after the promise of connection that seemed to come from the widespread temporal dislocation caused by the pandemic, becomes just another proof of one’s lack of belonging.

This trauma must be acknowledged and taken into account if we are to move forward. Both Frank and Toombs write about the necessity of crossing the temporal divide if proper care, medical and social, is to be provided. “To deny a chaos story,” Frank (1990, 109–10) writes, “is to deny the person telling this story, and people who are being denied cannot be cared for.” I do not claim that my story covers any experience but my own, but my hope is that using it as a case study for research regarding the temporal dimension of chronic mental illness will inspire further inquiry and that maybe others dealing with similar issues will find some small piece of it that resonates with them and makes them feel less alone. Living outside of *structured sequential narrative time* is painful and lonely. And while we all desire to eventually move out of the chaos and back into objective time, what we need first and foremost is witness. Frank (1990, 110) writes that “chaos is never transcended but must be accepted before new lives can be built and new stories are told.” This is part of my journey to accepting my chaos. I hope it can help others to begin to find a way through theirs.
